# Public Health Surveillance and Reporting for Human Toxoplasmosis — Six States, 2021

**DOI:** 10.15585/mmwr.mm7128a1

**Published:** 2022-07-15

**Authors:** Jayden McCall, Laura Rothfeldt, Kelly Giesbrecht, Amanda Hunt, Leah Bauck, Joni Scheftel, Rachael Birn, Bryan Buss, Betsy Schroeder, Thomas E. Haupt, Rachel Klos, Anne Straily

**Affiliations:** ^1^Department of Diagnostic Medicine/Pathobiology, College of Veterinary Medicine, Kansas State University, Manhattan, Kansas; ^2^Zoonotic Diseases Section, Arkansas Department of Health; ^3^Kentucky Department for Public Health; ^4^Infectious Disease Epidemiology, Prevention and Control Division, Minnesota Department of Health; ^5^Nebraska Department of Health and Human Services; ^6^Council of State and Territorial Epidemiologists, Atlanta, Georgia; ^7^Division of State and Local Readiness, Center for Preparedness and Response, CDC; ^8^Pennsylvania Department of Health; ^9^Wisconsin Department of Health Services; ^10^Division of Parasitic Diseases and Malaria, Center for Global Health, CDC.

Toxoplasmosis is caused by infection with the zoonotic parasite *Toxoplasma gondii*. Although disease tends to be mild (e.g., self-limiting influenza-like symptoms) or asymptomatic in immunocompetent persons, toxoplasmosis is more severe in immunocompromised persons, who can develop potentially fatal encephalopathy (*1*). In addition, primary infections acquired during pregnancy might result in a range of adverse outcomes, including fetal ocular infection, cranial and neurologic deformities, stillbirth, and miscarriage (*1*,*2*). An estimated 11% of the U.S. population aged ≥6 years are seropositive for toxoplasmosis, based on analysis of sera collected through the National Health and Nutrition Examination Survey during 2011–2014 (*3*). Toxoplasmosis is not a nationally notifiable disease in the United States, and currently no national public health surveillance data are available; however, it is reportable in eight states. To better understand how surveillance data are collected and used, reviews of state-level toxoplasmosis surveillance were conducted during June–July 2021 using semistructured interviews with health officials in six states (Arkansas, Kentucky, Minnesota, Nebraska, Pennsylvania, and Wisconsin) where toxoplasmosis is currently reportable. Why or when toxoplasmosis became reportable could not be determined, and many of the states had limited capacity to respond to reported cases. Case definitions varied considerably in terms of clinical description, laboratory criteria, and case classification (i.e., confirmed, probable, or suspect), limiting disease estimates and comparisons among states. Implementation of a standardized case definition would help ensure that cases are counted consistently, enabling better use of surveillance data to characterize disease. Identifying newly acquired cases is challenging because most acute cases among immunocompetent persons (including pregnant women) are asymptomatic, disease among immunocompromised persons is likely reactivation of latent disease, and congenital infections might not manifest until later in life.

Members of the family Felidae (cats) are definitive hosts for *T. gondii*. Humans can be infected through various routes, including fecal-oral contamination from cats; consumption of undercooked contaminated meat, contaminated unwashed fruits or vegetables, contaminated water, and unpasteurized milk; vertical transmission from an infected mother; and organ transplantation. Toxoplasmosis is likely underdiagnosed in the United States: approximately 90% of infections among immunocompetent persons are asymptomatic or nonspecific and self-limiting (*1*); when symptoms are present, they can potentially mimic other more frequently encountered illnesses, including influenza and tickborne-diseases such as Lyme disease or anaplasmosis; thus, physician awareness and clinical suspicion of toxoplasmosis might be low, resulting in delays in or missed opportunities for diagnosis and case identification (*4*,*5*). No national maternal toxoplasmosis screening program currently exists, and most infants born with congenital toxoplasmosis appear normal at birth (*1*,*2*). Because reactivation of toxoplasmosis during immunocompromise can be rapidly fatal, and outcomes of congenital infection can be severe, toxoplasmosis surveillance could help improve awareness and understanding of disease prevalence and transmission routes and identify opportunities for prevention and control.

States where toxoplasmosis is reportable were identified using the State Reportable Conditions Assessment query tool on the Council of State and Territorial Epidemiologists (CSTE) website* and supplemented by reviewing reportable diseases lists accessed from state health department websites for inclusion of toxoplasmosis. A modified version of a qualitative questionnaire used for Chagas disease surveillance (*6*) was developed to identify why toxoplasmosis was designated a reportable condition in the state, how cases are reported and by whom, what actions are taken after case identification, how surveillance data are used and disseminated, whether nonhuman data are collected and used, and whether formal toxoplasmosis maternal screening programs are in place (*6*). State public health veterinarians were contacted by email and invited to participate either by telephone or virtual interview or to complete the questionnaire. The purpose and scope of the project, as well as a copy of the questionnaire, were included in this initial email. This activity was reviewed by CDC and was conducted consistent with applicable federal law and policy.^†^

As of April 2021, toxoplasmosis is reportable in eight states (Arkansas, Delaware, Hawaii, Kentucky, Minnesota, Nebraska, Pennsylvania, and Wisconsin); among these states, public health personnel from six (Arkansas, Kentucky, Minnesota, Nebraska, Pennsylvania, and Wisconsin) agreed to participate and were interviewed. The historic dates when toxoplasmosis became reportable and the reasons for initiating surveillance could not be determined. Toxoplasmosis had been reportable for as long as could be remembered and reviewing historical case data could not further elucidate a starting date. Several possible reasons were suggested for why toxoplasmosis was made reportable, including monitoring disease prevalence, a need to identify the source of infection, the effect of toxoplasmosis on pregnancy, congenital transmission, and outbreak identification. In addition, whether any substantial changes had occurred in how surveillance data were collected since toxoplasmosis became reportable also could not be determined.

Case definitions were provided by the states and varied considerably in both clinical and laboratory criteria and how cases are classified (Table). Variations in clinical descriptions included a separate description for immunocompromised persons (all states except Arkansas and Minnesota); a separate description for infection acquired during pregnancy (Kentucky, Nebraska, and Wisconsin); further separation based on timing of infection during pregnancy (early versus late; Pennsylvania); and a category for chronic infection status (Minnesota). Signs and symptoms used in each clinical description category were similar but not consistent. Variations in laboratory criteria included whether paired or sequential antibody testing was confirmatory (all states except Arkansas and Pennsylvania); single antibody titers as presumptive/suggestive criteria (Arkansas and Kentucky); criteria for congenital infection in infants (Kentucky, Nebraska, and Wisconsin); and a criterion that included testing at a reference laboratory (Minnesota). Among the variations in case classification were that Nebraska and Wisconsin had only a “confirmed” case classification and no “probable” classification, Pennsylvania also included a “suspect” classification, and Minnesota’s case classifications specified “confirmed,” “probable,” and “chronic.”

**TABLE:** Toxoplasmosis surveillance case definitions — six states, 2021

State	Clinical description	Laboratory criteria	Case classification
Arkansas	Cervical lymphadenopathy and/or influenza-like illness and/or ocular infection with vision loss	Elevated *Toxoplasma gondii–*specific IgG, IgM, IgA, and/or IgE titers (presumptive)	Probable: a clinically compatible case or asymptomatic person* with laboratory results indicative of presumptive infection
		Isolation of *T. gondii* in blood/fluids; detection of tachyzoites in tissue; and/or detection using PCR (confirmatory)	Confirmed: a clinically compatible case with confirmatory laboratory results
Kentucky	Fever, lymphadenopathy, and/or lymphocytosis	Single antibody titer (suspect)	Probable: a clinically compatible illness that is laboratory suspect
	Immunocompromised persons: above, plus myocarditis, pneumonia, and/or cerebral signs	Significant change in paired specimen antibody titers; demonstration of *T. gondii* in tissues/fluids; detection by PCR; and/or specific IgM or increasing titer in sera in congenital infection (confirmed)	Confirmed: a clinically compatible illness that is laboratory confirmed; clinical diagnosis and laboratory confirmed
	Infection during pregnancy: congenital anomalies or infant mortality		
Minnesota	Influenza-like illness, fever, lymphadenopathy, ocular pain, chorioretinitis, encephalitis, other systemic manifestations	Positive IgM test with or without positive IgG test (without confirmation at reference laboratory) (probable)	Probable: a clinically compatible illness with positive IgM test and without confirmation at reference laboratory
		Demonstration of *T. gondii* in any tissue; or *T. gondii* diagnosis by ocular exam; or positive PCR; or positive IgM confirmed at reference laboratory; or low IgG avidity test (confirmatory)	Confirmed: a clinically compatible illness with any of the listed confirmatory laboratory criteria
		Positive IgG with negative or equivocal IgM; or positive IgG and positive IgM on screening but negative IgM by confirmatory test; or high IgG avidity (chronic)	Chronic: laboratory results indicative of infection acquired in the distant past
Nebraska	Fever, lymphadenopathy, malaise, myalgia, lymphocytosis, and/or elevated liver enzymes	Detection of *T. gondii* in tissue or by PCR; and/or IgG/IgM change in paired serology; in infants, demonstration of specific IgM or increasing titer in paired sera (confirmed)	Confirmed: a clinically compatible illness that is laboratory confirmed
	Immunocompromised: chorioretinitis, myocarditis, pneumonia, and/or encephalitis		
	Neonatal infection: fever, rash, jaundice, and/or chorioretinitis		
	Infection during pregnancy: infant death or congenital abnormalities		
Pennsylvania	Immunocompetent: lymphadenopathy and/or ocular infection (uveitis)	Sequential sera displaying fourfold rise in *T. gondii*–specific IgG antibody titer (supportive)	Probable: a case that meets the clinical case definition and has only supportive laboratory results
	Immunodeficient: encephalitic symptoms with or without pulmonary/cardiac involvement	Demonstration of *T. gondii* organisms in tissue; demonstration of tachyzoites in tissue by histopathology; and/or positive PCR (confirmatory)	Confirmed: a case that meets the clinical case definition and is laboratory confirmed
	Newborn infants (early pregnancy infection): fever, lymphadenopathy, microcephaly, megalocephaly, rash, and/or anemia		Suspect: a case that meets clinical case definition and has other laboratory testing, or no laboratory testing was performed
	Newborn infants (third trimester infection): ocular complications/developmental delays in later life		
Wisconsin	Fever, lymphadenopathy, and/or lymphocytosis	Change in paired specimen antibody titer; demonstration of *T. gondii* in tissues/fluids; detection by PCR; and/or specific IgM^†^ or increasing titer in sera in congenital infection (confirmed)	Confirmed: a clinically compatible illness that is laboratory confirmed
	Immunocompromised: above, plus myocarditis, pneumonia, and/or cerebral signs		
	Infection during pregnancy: congenital anomalies or infant mortality		

**Abbreviations:** Ig = immunoglobulin; PCR = polymerase chain reaction; *T. gondii* = *Toxoplasmosis gondii*.

* Asymptomatic persons with laboratory evidence of presumptive infection are counted as a probable case at the time of initial report.

^†^ Demonstration of IgM antibody in adults does not meet case definition.

After notification of a case, all states attempt to investigate to determine exposure and clinical history; however, investigation depends on resource availability. Laboratories are the primary reporting source in all states, although physicians might also report cases. No state reported having formal maternal screening programs for toxoplasmosis; however, maternal screening is frequently recorded as the reason for testing on case report forms submitted to the state health department (Minnesota). No states collected nonhuman data as a routine part of toxoplasmosis surveillance. The Nebraska state public health veterinarian indicated that their office receives data from the state veterinary diagnostic laboratory about toxoplasmosis diagnosed in animals. Although such reports contain the animal owner’s city and zip code, they do not name the owner and are not formally integrated with the human case surveillance program.

Dissemination of surveillance data occurs through public reports posted to state health department websites (Arkansas, Minnesota, and Wisconsin) or updates to toxoplasmosis case counts in annual disease tables available on the state health department website (Kentucky). In two states (Nebraska and Pennsylvania), reports are distributed internally within the agency, but not externally.

## Discussion

Standardized surveillance case definitions provide a common, accepted set of criteria to ensure that cases of disease are classified and counted consistently, irrespective of jurisdiction.^§^ Surveillance data provide an evidence base about disease prevalence, including who is affected, where, and how, to guide the development, implementation, funding, monitoring, and evaluation of disease control activities^¶^ (*7*).

 Important differences were identified in case classifications and laboratory and clinical criteria used in surveillance case definitions, making it difficult to compare case counts or disease prevalence among states. Toxoplasmosis poses unique challenges for public health surveillance, primarily in identifying acute illnesses, which are the more important target for public health action (e.g., identifying and mitigating the source of infection). Once infected, persons are presumed to remain infected for life (even with treatment**) and likely maintain detectable antibody levels, even without reverting to or showing signs of active disease (latent or chronic infections) (*1*). Toxoplasmosis among immunocompromised persons more commonly represents reactivation of latent infection rather than newly acquired infection (*1*). Congenital infections might not manifest until later in life. Commercially available serology assays, which typically examine immunoglobulin (Ig) G and IgM antibody levels, cannot reliably differentiate between acute and chronic infection: IgM antibodies might remain elevated for ≥18 months after infection (*8*), and IgG might be present during acute infections (*9*). A combination of advanced serologic tests, such as IgG avidity testing or IgA or IgE antibody levels, available only through a reference laboratory,^††^ are necessary to serologically differentiate between acute and chronic infection. Direct detection methods such as polymerase chain reaction or histologic examination of tissue sections or smears of body fluid are more definitive in demonstrating active infection but are most useful in immunocompromised patients (*1*).

The findings in this report are subject to at least two limitations. First, only six of the eight states that conduct toxoplasmosis surveillance participated in the assessment; case definitions or processes for toxoplasmosis surveillance in Delaware or Hawaii were not able to be reviewed. Because of the length of time toxoplasmosis has been reportable in these states, most historic questions could not be answered. Second, this evaluation was conducted during the SARS-CoV-2 B.1.617.2 (Delta) variant surge of the ongoing COVID-19 pandemic, which might have affected staff member availability to gather historical information on procedures for toxoplasmosis investigation and response.

Developing and implementing a standardized case definition in states where toxoplasmosis is reportable could help ensure that surveillance data are collected in a standardized way and establish common goals for surveillance. As a result of this review, the participating states have decided to proactively develop a CSTE position statement for a standardized surveillance case definition for toxoplasmosis.

**Summary**

**What is already known about this topic?**

Toxoplasmosis, a zoonotic parasitic disease that can result in severe adverse outcomes, is not a nationally notifiable illness in the United States; no national level surveillance data are available.

**What is added by this report?**

In 2021, toxoplasmosis was reportable in eight states. Among six states that participated in a surveillance evaluation, case definitions varied considerably, and a need for development and implementation of a standardized case definition was identified.

**What are the implications for public health practice?**

Implementing a standardized case definition would help ensure that cases are counted consistently. Toxoplasmosis surveillance could increase awareness among physicians and public health personnel but is dependent upon health department resources. Identifying newly acquired cases is important for surveillance but is challenging because most acute cases among immunocompetent persons (including pregnant women) are asymptomatic, disease among immunocompromised persons are likely reactivations of latent disease, and congenital infections might not manifest until later in life.

## Footnotes

*
https://www.cste.org/group/SRCAQueryRes

† 45 C.F.R. part 46; 21 C.F.R. part 56.

§ https://ndc.services.cdc.gov/ (Accessed March 2, 2022).

¶ https://www.cdc.gov/nndss/about/index.html (Accessed March 2, 2022).

** https://www.cdc.gov/parasites/toxoplasmosis/prevent.html; https://www.cdc.gov/parasites/toxoplasmosis/health_professionals/index.html

††
https://www.sutterhealth.org/services/lab-pathology/toxoplasma-serology-laboratory
